# Autoimmune enteropathy: not all flat mucosa mean coeliac disease 

**Published:** 2016

**Authors:** Umberto Volta, Maria Gloria Mumolo, Giacomo Caio, Elisa Boschetti, Rocco Latorre, Fiorella Giancola, Paola Paterini, Roberto De Giorgio

**Affiliations:** 1 *Department of Medical and Surgical Sciences, University of Bologna, Italy*; 2 *Gastroenterology Unit, Department of Gastroenterology, University of Pisa, Italy*

**Keywords:** Villous atrophy, Malabsorption, Enterocyte autoantibodies, Autoimmune enteropathy

## Abstract

A 62-year-old woman complaining of severe malabsorption was diagnosed with celiac disease based on the findings of flat, small intestinal mucosa and HLA-DQ2 positivity, although celiac serology was negative. This diagnosis was questioned due to the lack of clinical and histological improvement after a long period of strict gluten-free diet. The detection of enterocyte autoantibodies guided to the correct diagnosis of autoimmune enteropathy, leading to a complete recovery of the patient following an appropriate immunosuppressive treatment. Autoimmune enteropathy should be considered in the differential diagnosis of malabsorption with severe villous atrophy, including those cases with negative celiac-related serology.

## Introduction

 Autoimmune enteropathy (AIE) is a rare disorder characterized by chronic diarrhea, small intestinal atrophy and circulating enterocyte autoantibodies (EAA) (1). Although AIE is more prevalent in infants with an estimated incidence of <1 in 100.000 newborns (2), this immune-mediated disorder has been reported also in adult age (1, 3-5). Due to its autoimmune origin, this clinical entity displays a frequent association with other immune-mediated disorders, e.g. autoimmune thyroiditis (3).

Due to clinical and histopathological features of AIE mimicking celiac disease (CD), patients are usually misdiagnosed and treated by gluten-free-diet (GFD) without any improvement. Left untreated (or with inappropriate treatment), AIE can evolve to major and life-threatening complications, such as severe malabsorption and intestinal failure. Thus, a timely diagnosis is mandatory to start an appropriate immunosuppressive regimen, which is the gold standard therapeutic strategy for patients with AIE.

Herein, we report the case of a 62-year-old woman complaining of severe malabsorption with untreatable diarrhea and severe villous atrophy unresponsive to GFD. The finding of EAA led to the diagnosis of AIE with significant clinical improvement after immunosuppressive treatment. 

## Case Report

**Figure 1:**
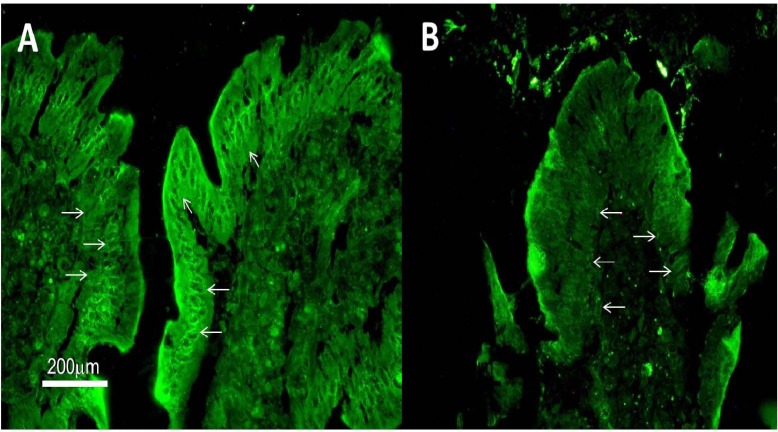
Representative photomicrographs illustrating positive enterocyte autoantibodies (EAA) resulting from indirect immunofluorescence on monkey jejunum previously incubated with the patient’s serum. Both pictures (A) and (B) show IgG and IgA antibody pattern (final dilution: 1:400 and 1:100, respectively) characterized by a bright staining of enterocyte cytoplasm (arrows) with negative nuclei along with a positivity of the brush border / microvilli (calibration bar: 200 μm in A and B

A 62-year-old female patient had an unremarkable clinical history until the age of 41, when a diagnosis of autoimmune thyroiditis with euthyroidism was established. At age of 47, she experienced bilateral ptosis, diplopia, weakness of masticatory muscles and generalized fatigability involving four limbs. Diagnosis of myasthenia gravis (MG) was posed on the basis of a positive repetitive nerve stimulation test and circulating anti acetylcholine-receptor antibodies. MG was found to be associated with a thymoma treated with thymectomy. At age of 59, due to onset of acute diarrhea (up to 10 bowel movements / day) with fecal incontinence, the colon-ileoscopy of the patient showed a normal large bowel and terminal ileum mucosa. A summary of the most relevant clinical, laboratory and histopathological findings has been reported in Table 1. A few months later, the patient experienced worsening of the diarrhea (up to 15 bowel movements / day) with abdominal pain, weight loss (about 8 kg in the last 6 months), muscle cramps and numbness. Blood tests revealed low levels of Vitamin D3, serum albumin, and electrolytes (i.e., Ca++, Mg++, K+). Serological tests for celiac disease (i.e., IgA tissue transglutaminase – tTGA; IgA endomysial – EmA; and IgG deamidated gliadin antibodies - DGP) were negative. Total serum IgA levels resulted in the normal range. Screening for immune markers showed a positive result for antinuclear antibodies on HEp2-cells (ANA 1:80 with a nucleolar pattern). Due to the persistence of severe malabsorption syndrome, the patient underwent an upper gastrointestinal endoscopy with duodenal biopsy, which revealed subtotal villous atrophy (grade III according to Marsh classification) (6). Genetic testing was consistent with CD diagnosis and showed positive HLA-DQ2 in heterozygosis. Based on the histopathological and genetic findings, the patient was diagnosed as seronegative CD and started GFD. Despite adherence to gluten withdrawal, her condition continued to worsen due to persistent diarrhea and significant laboratory abnormalities, i.e. low levels of albumin, folic acid, Mg++ and K+. The lack of response to GFD was attributed to an inadvertent gluten contamination since the patient was a baker. The patient was hospitalized in the Department of Gastroenterology of the University of Pisa, because symptoms (diarrhea, nausea and anorexia) did not resolve. On admission, physical examination revealed a malnourished patient (BMI = 19) and laboratory tests showed a macrocytic anemia (Hb 11.5 g / dL), low levels of folic acid, vitamin D3, albumin and Mg++. Due to lack of clinical and laboratory responses to GFD, an upper gastrointestinal endoscopy was performed to check the status of small intestinal mucosa. The duodenal biopsy documented a persistent subtotal villous atrophy with crypt hyperplasia and increased number of IELs (40%-60%), thus challenging the diagnosis of CD. Other causes of villous atrophy, including: parasitic infection (*Giardia lamblia*), small intestine bacterial overgrowth, immunodeficiencies, eosinophilc gastroenteritis, drug-induced enteropathy (related to non-steroidal anti-inflammatory drugs and angiotensin II inhibitors) and Whipple disease were excluded by appropriate investigations. The patient was re-hydrated and treated with parenteral nutrition, electrolyte supplementation and albumin; gluten was reintroduced in the diet. Following a partial improvement (mild weight increase), the patient was discharged. However, she was re-admitted in the same Gastroenterology unit few months later due to sudden deterioration characterized by hypocalcaemia-related tetany, mild macrocytic anemia, low serum levels of Mg++ and severe hypoalbuminemia with mild ascites confirmed by CT scan. Serology for CD (EmA, tTGA, DGP) was confirmed to be negative. Apart from ANA and anti-acetylcholine-receptor antibodies, a thorough autoimmune profile, including: non-organ specific (smooth muscle, mitochondrial and liver-kidney microsomal) and organ specific (gastric parietal cells, glutamate decarboxylase, neurohypophysis) antibodies were negative. EAA, performed in the Immunology Laboratory of the Bologna University, disclosed positivity for IgG and IgA antibodies at the titer of 1:400 and 1:100, respectively (Fig. 1). Due to this finding, supporting the diagnosis of autoimmune enteropathy, the patient started methylprednisolone (0.5 mg/kg/day) and azathioprine (50 mg/day for 7 days and then increased up to 100 mg/day) and showed a rapid significant clinical improvement. Diarrhea resolved in 15 days and the patient gained weight progressively until reaching a normal BMI in a few months. Laboratory findings progressively normalized due to the improved mineral and vitamin absorption. After 6 months of immunosuppressive treatment, EAA IgA antibodies were negative, whereas EAA IgG antibodies persisted positive at a reduced titer (1:100). Methylprednisolone was progressively tapered down to 4 mg/day, whereas azathioprine was replaced by 6-mercaptopurine due to vomiting. The patient’s clinical conditions are steadily improving with a complete remission of symptoms and weight gain after six months of follow-up. 

**Table 1:** Clinical, laboratory and histological data of the patient during follow-up

	December 2012	May 2013	January 2014	December 2014	May 2015	November 2015
Symptoms	Diarrhea 10 times/day	Diarrhea 15 times/day	Diarrhea 10 times/day	Diarrhea 10 times/day	Diarrhea 10 times/day	Normal bowel movements
Body mass index	25.5	22.6	20.5	19.0	20.7	21.5
Hemoglobin 12-16 g/dL	13.9 g/dL	13.2 g/dL	13.0 g/dL	11.5 g/dL	11.1 g/dL	13.9 g/dL
Albumin 3.5-5.2 g/dL	2.8 g/dL	2.6 g/dL	3.0 g/dL	2.8 g/dL	2.5 g/dL	3.8 g/dL
Folic acid 4.6-18.7 ng/mL	not done	5.6 ng/mL	4.5 ng/mL	4.0 ng/ml	not done	15.1 ng/mL
Ferritin 15-180 mg/dL	not done	85 mg/dL	61 ng/mL	not done	64 ng/mL	68 ng/mL
Vitamin D 30-100 ng/mL	28.0 ng/mL	26.0 ng/mL	not done	15.8 ng/mL	not done	49.0 ng/mL
K^+^ 3.5-5.3 mEq/L	2.0 mEq/L	3.1 mEq/L	2.9 mEq/L	3.6 mEq/L	3.9 mEq/L	4.2 mEq/L
Mg^++^ 1.5-2.6 mEq/L	not done	1.0 mEq/L	2.0 mEq/L	1.7 mEq/L	1.0 mEq/L	2.2 mEq/L
Ca^++^ 8.4-10.2 mg/dL	8.4 mg/dL	7.3 mg/dL	8.4 mg/dL	8.5 mg/dL	7.5 mg/dL	9.2 mg/dL
EmA IgA	negative	negative	negative	negative	negative	negative
tTGA IgA	negative	negative	negative	negative	negative	negative
DGP IgG	negative	negative	negative	negative	negative	negative
EAA IgA	not done	not done	not done	not done	positive 1:100	negative
EAA IgG	not done	not done	not done	not done	positive (1:400)	positive (1:100)
ANA HEp-2 cells	not done	positive (1:80; nucleolar pattern)	not done	not done	positive (1:80; nucleolar pattern)	not done
HLA typing	not done	DQ2 (DQA105, DQB1 02)	---	---	---	---
Duodenal biopsy	not done	Subtotal villous atrophy (Marsh III)	not done	Subtotal villous atrophy (Marsh III)	not done	not done
Diagnosis	Unclassified malabsorption	Celiac disease	Celiac disease	Unclassified malabsorption	Autoimmune enteropathy	Autoimmune enteropathy
Treatment	Anti-diarrheal drugs	Gluten free diet	Gluten free diet	Gluten containing diet	Methylprednisolone 0.5mg/kg/day plus azathioprine 100 mg/day	Methylprednisolone 4 mg/day plus 6-mercaptopurine 50 mg/day

## Discussion

This case report indicates that patients with a flat, small bowel mucosa and negative celiac serology should not be necessarily labeled as seronegative CD. Indeed, several aspects are essential for an *a-posteriori* analysis: 1) the lack of response to GFD; 2) the progressive worsening with weight loss and persistent signs of intestinal malabsorption; 3) the exclusion of a number of conditions responsible for flat small bowel mucosa; and 4) the detection of AIE biomarkers, i.e. EAA, finally guiding to a correct diagnosis. Taken together, these peculiar features ruled out the CD and provided the basis for addressing physicians and gastroenterologists to consider AIE in the diagnostic work-up of CD-seronegative patients with a flat intestinal mucosa.

AIE is a rare, although very severe disorder, causing profound immune-mediated changes of the intestinal mucosal structure and function, progressing to complications such as severe malabsorption and irreversible intestinal failure. As reported in most patients with AIE (1, 3), our patient displayed a close association with autoimmune disorders (i.e., MG and autoimmune thyroiditis) and positivity for immune markers (i.e., ANA, thyroid antibodies, and anti-acetylcholine-receptor antibodies). Serum EAA can be considered as a specific marker of AIE, being consistently negative in patients with other autoimmune intestinal and extraintestinal disorders although they lack sensitivity (4, 7). In fact, Akram et al. showed these autoantibodies in patients with AIE, thus suggesting sensitivity not higher than 60% (3). The EAA belongs mainly to IgG and IgA class and the antibody titer usually decreases after immunosuppressive treatment as observed in this reported patient (4, 5). Other autoimmune markers, such as anti-goblet cell antibodies, have been reported in AIE patients, but their specificity for AIE is poor since they have been detected in patients with inflammatory bowel disease as well (8).

The pathophysiology of AIE is far to be understood, but it is likely that this disorder recognizes an underlying defect in regulatory T-cell system. Although the EAA triggers a cascade of mechanisms involving the complement fixation to cells, it is generally acknowledged that EAA represents an immunologic epiphenomenon since they do not exert a pathogenic role in AIE (9). Due to its frequent association with other autoimmune disorders, AIE has been included in the IPEX (Immunodysregulation Polyendocrinopathy Enteropathy X-linked) or APECED (Autoimmune Phenomena, Polyendocinopathy, Candidiasis, and Ectodermal Dystrophy) syndromes (10). In this line, the a link between AIE and MG / thymoma, also detectable in the clinical history of our case, represents a peculiar association which confirms and expands previously published data (11-13).

The positivity of HLA-DQ2 along with the high number of IELs and the persistence of flat mucosa after GFD raise the possibility that our patient might have a refractory CD (RCD) accompanying AIE. Although we cannot rule out a coexistent RCD, the association between these two conditions is very rare (14). Notably, it should be underlined that about 20% of patients with AIE display the same immunohistochemical and genetic features of CD (3). A peculiar histopathological finding which distinguishes AIE from RCD is the number of / immunopositive lymphocytes, which are always increased in CD, while they are very low in AIE (4). However, this test was not performed in our patient. Therefore, we cannot draw conclusions on this important diagnostic immunohistochemical technique. Concerning genetics, the prevalence of HLA DQ2, a well-established CD marker, is also quite high in AIE, particularly in those cases with a frequent association with other autoimmune disorders, as observed in our patient (3).

Finally, the outcome of patients with AIE is dependent upon an early recognition and treatment of the disorder, which requires immunosuppressive drugs. Since the severe malabsorption underlying AIE can be fatal if not timely treated, it is mandatory to start the therapy as soon as possible. In our patient, the response to steroid and immunosuppressive therapy resulted in a rapid and significant improvement of the general conditions with the disappearance of diarrhea and malabsorption signs.

In conclusion, the analysis of the present case highlights that AIE should be considered in the differential diagnosis of malabsorption with severe villous atrophy and the negativity of CD serology. The positivity for EAA is important to confirm the diagnosis of AIE. Rapid immunosuppressive treatment can significantly improve the outcome of patients with AIE.

## Note

**(**Please cite as:** Volta U, Mumolo MG, Caio G, Boschetti E, Latorre R, Giancola F, et al. ****Autoimmune enteropathy: not all flat mucosa mean coeliac disease. Gastroenterol Hepatol ****Bed Bench**** 2016;9(2):140-145).**

## References

[1] Corazza GR, Biagi F, Volta U, Andreani ML, De Franceschi L, Gasbarrini G (1997). Autoimmune enteropathy and villous atrophy in adults. Lancet.

[2] Masia R, Peyton S, Lauwers GY, Brown I (2014). Gastrointestinal biopsy findings of autoimmune enteropathy: a review of 25 cases. Am J Surg Pathol.

[3] Akram S, Murray JA, Pardi DS, Alexander GL, Schaffner JA, Russo PA (2007). Adult autoimmune enteropathy: Mayo Clinic Rochester experience. Clin Gastroenterol Hepatol.

[4] Carroccio A, Volta U, Di Prima L, Petrolini N, Florena AM, Averna MR (2003). Autoimmune enteropathy and colitis in an adult patient. Dig Dis Sci.

[5] Volta U, De Angelis GL, Granito A, Petrolini N, Fiorini E, Guidi M (2006). Autoimmune enteropathy and rheumatoid arthritis: a new association in the field of autoimmunity. Dig Liver Dis.

[6] Marsh MN, Johnson MW, Rostami K (2015). Rebutting Oberhuber- Again. Gastroenterol Hepatol Bed Bench.

[7] Mirakian R, Richardson A, Milla PJ, Walker-Smith JA, Unsworth J, Savage MO (1986). Protracted diarrhoea of infancy: evidence in support of an autoimmune variant. Br Med J.

[8] Hibi T, Ohara M, Kobayashi K, Brown WR, Toda K, Takaishi H (1994). Enzyme linked immunosorbent assay (ELISA) and immunoprecipitation studies on anti-goblet cell antibody using a mucin producing cell line in patients with inflammatory bowel disease. Gut.

[9] Ruemmele FM, Brousse N, Goulet O (2004). Autoimmune enteropathy: molecular concepts. Curr Opin Gastroenterol.

[10] Gentile NM, Murray JA, Pardi DS (2012). Autoimmune enteropathy: a review and update of clinical management. Curr Gastroenterol Rep.

[11] Montalto M, D'Onofrio F, Santoro L, Gallo A, Gasbarrini A, Gasbarrini G (2009). Autoimmune enteropathy in children and adults. Scand J Gastroenterol.

[12] Mais DD, Mulhall BP, Adolphson KR, Yamamoto K (1999). Thymoma-associated autoimmune enteropathy. A report of two cases. Am J Clin Pathol.

[13] Elwing JE, Clouse RE (2005). Adult-onset autoimmune enteropathy in the setting of thymoma successfully treated with infliximab. Dig Dis Sci.

[14] Valitutti F, Barbato M, Aloi M, Marcheggiano A, Di Nardo G, Leoni S (2014). Autoimmune enteropathy in a 13-year-old celiac girl successfully treated with infliximab. J Clin Gastroenterol.

